# Pathogenesis of interstitial lung disease in systemic sclerosis

**DOI:** 10.2478/rir-2024-0020

**Published:** 2024-10-21

**Authors:** Nina Goldman, Voon H Ong, Christopher P. Denton

**Affiliations:** Center for Rheumatology, University College London, London, UK

**Keywords:** systemic sclerosis, interstitial lung disease, fibrosis, pathogenesis

## Abstract

Interstitial lung disease (ILD) is a frequent important complication of systemic sclerosis (SSc). Factors relevant to aetiopathogenesis of SSc are also central to SSc-ILD. Severity of SSc-ILD is variable but it has a major impact on morbidity and mortality. Factors determining SSc-ILD susceptibility reflect the genetic architecture of SSc and are increasingly being defined. There are aspects linked to immunogenomics and non-immunological genetic factors that may be less conserved and underlie some of the geographical and racial diversity of SSc. These associations may also underlie important links between autoantibody subgroups and patient level risk of SSc-ILD. Examination of blood and tissue samples and observational clinical research together with integrated analysis of *in vitro* and *in vivo* preclinical models have elucidated pathogenic mechanisms of SSc-ILD. These have confirmed the potential importance of immune mechanisms in the innate and adaptive immune systemic as well as a significant role for profibrotic pathways especially transforming growth factor beta (TGFbeta) and its regulators and downstream mediators. Recent analysis of clinical trial cohorts as well as integrated and multilevel high dimensional analysis of bio-samples has shed further light on SSc-ILD. This is likely to underpin future advances in stratified and precision medicine for treatment of SSc.

## Introduction

Systemic sclerosis (SSc) is a rare disease affecting around 1 in 10,000 individuals in most European countries.^[1]^ It typically occurs in adults between 40 and 60 years old however it can develop at any age, although estimated prevalence is much lower in children at 3 per 1, 000, 000 in the USA.^[2]^ Like many autoimmune diseases there is a female predisposition. That SSc develops with this rarity suggests that the factors causing SSc occur infrequently. Like most complex chronic diseases, the aetiopathogenesis is likely to be multifactorial and involve host susceptibility and environmental triggers.^[3]^

There has been considerable progress in understanding and defining pathogenic mechanisms over the past 3 decades. Lung involvement is the commonest cause of death in SSc, a condition with high case specific mortality of around 50%.^[4]^ Lung complications include pulmonary vascular disease and interstitial lung disease (ILD), the latter is the focus of this article.

ILD develops frequently within SSc but to differing extents and with variable clinical impact. Cohort studies show that around half of cases of diffuse cutaneous SSc (dcSSc) and a quarter of those with limited cutaneous (lcSSc) involvement develop definite ILD during their disease course.^[5]^ However, since lcSSc is much more common than dcSSc the two major skin subsets are roughly evenly distributed in an SSc-ILD cohort.^[6]^

## Overlapping Pathogenic Mechanisms

There are parallels between SSc-ILD and the overall disease in considering aetiopathogeneses that reflect susceptibility to ILD and triggering events that are likely to be intrinsic to the disease as well as environmental. There are notable associations between clinical phenotype, laboratory features of SSc and the development of SSc-ILD and its progression and severity. These factors give important insights into pathogenic mechanisms.

**Figure 1:**
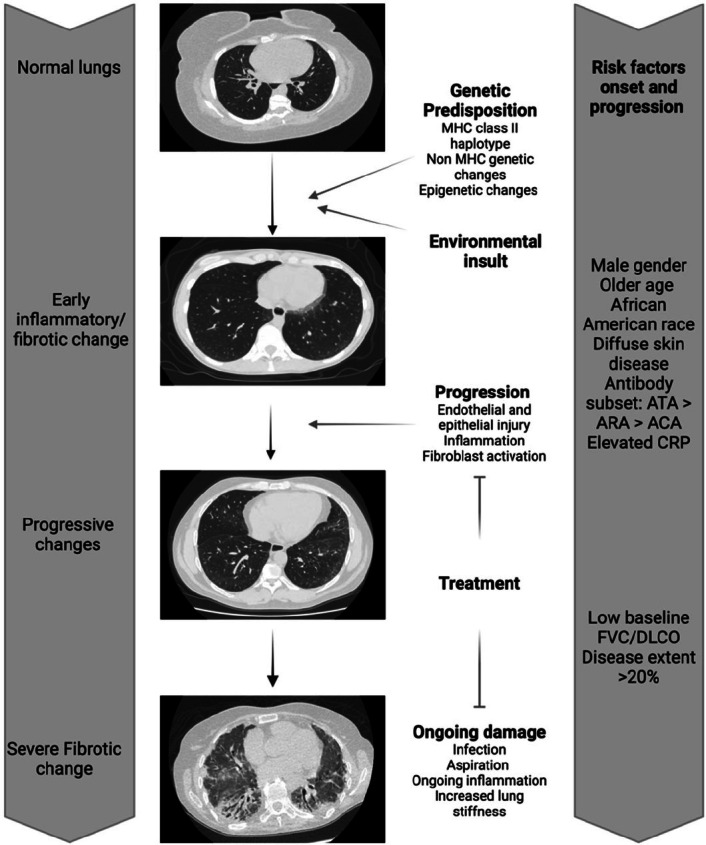
Overview of the pathogenesis of SSc in relation to ILD. Genetically predisposed individuals likely undergo an environmental trigger resulting in endothelial and epithelial injury. This triggers an inflammatory response and fibrosis. Ongoing injury and inflammation along with other factors such as infection, aspiration and increased lung stiffness lead to progression of fibrotic change. (Figure created with BioRender.com).

A general schematic summarising the development of SSc-ILD is provided in Figure 1. This integrates likely genetic, environmental and pathobiological aspects of the disease. These determine not only the development of ILD but also its severity and rate of progression.

## Preclinical Models

The prototypic mouse models of SSc were type 1 and type 2 tight skin (Tsk) mouse strains. These are now characterised as germline mutations of fibrillin-1 and collagen 3a1.^[7,8]^ The former leads to a gain of function transforming growth factor beta (TGFbeta) dependent fibrosis in the skin, especially the hypodermis, but neither strain develops lung fibrosis. In fact, the Tsk1 has features of emphysema. However, chemically and immunologically induced mouse models do show features of ILD.

The most widely studied mouse model is the bleomycin model that can be induced by surgical intratracheal or oropharyngeal gavage.^[9]^ It can also be seen after subcutaneous delivery of bleomycin but is less severe. Pulmonary delivery leads to marked inflammation from 5–7 days followed by fibrosis from 10–14 days. There is generally resolution of the lung fibrosis with restoration of normal lung architecture by 60 days. This model has been used to test antifibrotic agents although it is appreciated that it really reflects an acute lung injury model.

Immunological mechanisms are implicated in the mouse model of topoisomerase-1 immunisation and supports the potential for an immune response against topoisomerase-1 promoting or triggering fibrosis.^[10]^

The targeted genetic mouse strains have offered more detailed and precise insight and allowed testing of hypotheses of pathogenesis *in vivo*. The Fra-2 transgenic strain develops lung fibrosis as well as abnormalities in other organs relevant to SSc.^[11,12]^ Fibroblast directed activation of TGFbeta signalling confirms the importance of TGFbeta pathways in driving fibrosis. This constitutively active receptor expression led to lung and skin fibrosis.^[13]^ Likewise, fibrosis was seen in mice in which connective tissue growth factor (CTGF) had been activated using transgenic overexpression.^[14]^ Mice with postnatal deletion of type 2 TGFbeta receptor show no lung fibrosis in response to bleomycin and highlight the importance of resident lung fibroblasts in coordinating the fibrotic mechanisms in this experimental model.^[15]^

A mouse strain that replicates many aspects of SSc is the transgenic strain that has expression of a nonsignaling type 2 TGFbeta receptor on fibroblasts.^[16]^ This leads to an imbalance of signalling TGFbeta receptors and ligand dependent TGFbeta pathway activation.^[17]^ Mice are prone to excess fibrosis in response to minor tissue injury and this is relevant to the lung. Some spontaneous fibrosis develops but this is much more severe after instillation of unbuffered normal saline into the trachea. This represents a mildly acidic solution perhaps analogous to chronic gastro-oesophageal reflux in SSc and exemplifies potential mechanisms for initiation or amplification of fibrosis in susceptible cases of SSc. Interestingly this mouse strain has been a platform for a study showing that bleomycin induces a more severe and persistent fibrosis without the normal recovery of lung. This reflects less epithelial cell regeneration and proliferation after injury and persistence of myofibroblasts in the lung that leads to fibrosis persisting beyond 60 days.^[17]^

Together, these mouse stains have provided powerful insight into the development of SSc-ILD. They suggest that lung injury, including epithelial injury is important especially for persistent progressive lung disease along with immunological mechanisms. The notion is that systemic effects of SSc make tissue more susceptible to fibrosis in response to minor injury and that there may be fibroblast-dependent dysregulated connective tissue repair with scarring as a unifying disease mechanism.

Lanifibranor which is showing promising results and under clinical development as a potential treatment for liver fibrosis, was able to attenuate lung fibrosis induced by bleomycin in the transgenic mouse TbetaRIIDk-fib.^[18]^ However it was not effective in a well conducted phase 2 clinical trial in dcSSc. Similarly, the effect of nintedanib on dermal fibrosis in Fra-2 murine model was not evident in phase 3 clinical study in SSc.^[19]^ This example demonstrates the challenges of experimental therapeutic studies using these preclinical platforms as in general the lung fibrosis is more amenable to treatment in mice than in human SSc. A summary of pre-clinical models of is presented in Table 1.

**Table 1:** Key genetic factors linked to systemic sclerosis interstitial lung disease (SSc-ILD) or SSc antibodies

Gene	Gene function	SNP/Genotype		Relevant associated features	Reference
**Non-HLA related genes**					
CTGF	Synthesis extracellular matrix	rs6918698 / GG genotype or G allele		Associated ATA & ILD but not replicated in other studies	[14,15,16,17,22]
IRF5	Innate immune response	rs2004640		Inconsistently associated with ILD	[13,22]
STAT4	Adaptive immune response	rs7574865		T allele protective against ILD	[13,22]
IRAK1	Innate immune response	rs1059702		Inconsistently associated with ILD	[13,22,23]
MUC5B	Gel forming mucin	rs35705950		Not associated with SSc-ILD, associated with idiopathic pulmonary fibrosis	[21]
NLRP1	Assembly of inflammasome	rs8182352		Associated ATA & ILD	[24]
IL1A	Proinflammatory cytokine	CTG/CTG diplotype		Associated ILD	[25]
HGF	Anti-fibrotic factor and promotes angiogenesis	TT genotype		Associated end stage ILD no association between all SSc-ILD and SSc	[26]
*CD226*	Lymphocyte function	rs763361		Not associated with ILD	[13,22,24]
**HLA related genes**					
*HLA Class I*	Adaptive immune system function	B*62 CW*06:02 C*16:01 B*44:03		Associated ILD Associated ILD Protective against SSc Protective against SSc	
*HLA Class II*	Adaptive immune system function	DR		ACA	
			DRB1*01:01DRB1*08:01	ACA	
			DRB1*07:01	ACA	
			DRB1*11:01	ATA	
			DRB1*11:04	ATA	
			DRB1*15:01	ATA	[27,28]
			DRB5*01:01	ATA but not risk of SSc	
			DRB5*01:05 DRB4*01:01	ILD Protective against SSc	
				ACA	
		*DP*	DQA1*01:01 DQB1*05:01	ACA	
			DQB1*03:01	ATA	
			DQA1*02:01	LcSSc	
			DQA1*05:01 DQB1*02:02	DcSSC SSc	
		*DP*	DPA1*02:01	ATA but not risk of SSc	
			DPB1*13:01	SSc/ATA	

## Epidemiological Studies

SSc is rare and epidemiological studies have been challenging. A variety of methodologies has been used including hospital-based cohort analysis as well as attempts at population-based studies.^[20]^ The hospital-based series have tended to generate higher disease frequency possibly due to intrinsic referral bias. Whilst, population-based analyses risk underreporting and may underestimate true disease frequency, especially for less severe cases.^[21, 22, 23]^

Another limitation of many epidemiological studies are that different methods have been used to define presence of ILD. chest X-ray (CXR) is known to be a very poor screening tool. Recent studies comparing computed tomography (CT) with pulmonary function tests (PFTs) at the thresholds typically used to define the presence of ILD have also demonstrated the weaknesses of PFTs alone with high rates of false negative in detecting ILD.^[24]^ Thus, CT scan is the gold standard for detecting ILD and for this reason is now recommended as standard of care assessment for baseline evaluation of all SSc cases. Progressive disease is often defined using combination of PFTs, CT imaging and clinical symptoms.^[25]^

However, epidemiological studies give important clues relevant to the role of environment and host susceptibility in SSc-ILD pathogenesis.^[21]^ There is little evidence of clustering of cases that would support strong environmental triggers. Geographical and ethnic differences observed point toward potential genetic and epigenomic factors. These factors link to immunogenomics which are considered in more detail below and may overlap with other autoimmune rheumatic diseases.

## Genetic Susceptibility for SSc-ILD

There is robust evidence of genetic factors impacting on susceptibility to SSc-ILD. These go beyond the genetic factors linked to disease susceptibility that have now been well defined for common variant single nucleotide polymorphism (SNPs) using association studies including genome wide association study (GWAS) meta-analysis. Further work has explored potential links with rare variants that are more likely to have a direct functional role in SSc-ILD development. However, many genetic factors associated with ILD or ILD risk factors are not replicated in validation studies or meta-analysis.

The strongest genetic association with ILD is linked to antinuclear autoantibody (ANA) subgroup. Studies have confirmed that ANA reactivity links closely to class II major histocompatability complex (MHC) haplotype. Thus, anti-topoisomerase antibody (ATA) with high risk of ILD and other antibodies that associate with minimal risk of ILD such as anti-centromere antibody (ACA) have different associations.

Other genetic factors have been identified. STAT4 polymorphisms can be protective from SSc-ILD development.^[26]^ A functional polymorphism in the CTGF gene promoter provided a compelling mechanistic link when it was described as associated with SSc-ILD in a well-defined single centre cohort.^[27]^ Two studies did confirm the link however other studies have not replicated a generalised association and so it is likely that other genetic or non-genetic factors are also important.^[28,29,30,31]^ Epigenetic changes have been demonstrated on regulatory regions in immune cells in SSc and likely play a role in SSc-ILD pathogenesis.^[32]^ Genetic factors have also been identified that differentiate SSc from idiopathic pulmonary fibrosis highlighting the differences in pathogenesis of these diseases.^[33,34]^

As in other contexts association studies have raised strong candidate pathways and mediators relevant to pathogenesis, but it is very unlikely that a dominant genetic basis will be identified considering the complex aetiopathogenesis and lack of prominent heritability in SSc and SSc-ILD. A summary of genetic factors linked to SSc-ILD is provided in Table 2.

**Table 2:** Summary of pre-clinical models of SSc

Mouse model	Induction model	ILD	Inflammation	Auto-anti-bodies	Reference
**Genetic models**					
Tsk-1	Fibrillin 1 mutation	No	No	Fibrillin-1	[30]
Tsk-2	Collagen 3a-1 mutation	No	+	ACA, ATA	[31]
Fra-2	Overexpression Fra-2	Yes	++	No	[34]
CTGF	Overexpression CTGF in fibroblasts	Yes	Not reported	Not reported	[37]
TbetaRI	Postnatal induction TGFbetaRI	No	No	Not reported	[36]
TbetaRIIDk-fib	Expression kinase-deficient TbetaRIIDk-fib in fibroblasts	Yes	Not reported	Not reported	[39]
**Inducible models**					
Bleomycin	Subcut or Intratracheal bleomycin	Yes	++ (peaks between 3-5 days)	No	[32]
Hypochlorous	Injection hypochlorous acid	Yes	+ (in dermis)	Low level ATA	[43]
DNA Topoisomerase I	Injection DNA Topoisomerase I and CFA	Yes	++ (peaks around 8 weeks)	ATA	[33]
Sclerodermatous GVHD	Injection spleen cells into RAG-2 mice	Yes	++	ATA	[44]

## Lessons from Observational Cohort Studies

Observational cohort studies have been valuable in gaining better understanding of the timing and frequency of development of ILD in SSc. There appear to be two distinct but overlapping patterns of SSc-ILD. Elevated at-risk auto-antibodies such as ATA associate with a very high frequency of SSc-ILD developing within the first 3 years of disease.^[6]^ This is seen in both dcSSc and lcSSc with ATA reactivity.^[6]^ Other antibodies also seem to give increased risk of this early phase of SSc-ILD. Conversely some important antibodies such as anti-RNA polymerase III (ARA) are not associated with early significant SSc-ILD but may develop the complication at a later stage in disease.

Interestingly, the late-stage lung fibrosis and progression appears to link more with the disease cutaneous subset so that group level decline is more in lcSSc than dcSSc. This later phase may be less immunologically driven and reflect pathogenic mechanisms that are more like IPF including chronic epithelial injury, feed forward activation of profibrotic cells and profibrotic senescent fibroblasts. However, dysregulated immune responses are reported in IPF with elevated circulating plasmablasts in these patients. Immune activation may remain operational in late stage SSc ILD and presence of abnormal lymphoid aggregates in fibrotic lung is supportive of this.^[35,36]^ Lung stiffness could also be a relevant contribution, and this may explain that fibrosis progression associates with the extent of ILD on CT scan.^[37]^

## Experimental Medicine Studies and Clinical Trials

Recent clinical trials have been very informative about pathogenic mechanism and have reinforced views about the links between immuno-inflammation and development of SSc-ILD. They also support the concept that early progressive ILD in high-risk patients might have distinct drivers and mechanisms compared to more established later stage SSc-ILD.

An important consideration however is the clinical trial populations studies. For example, the trials of immunosuppression have focused on disease with ground glass on CT and perhaps earlier stage cases. This may explain that treatment with cyclophosphamide (CYC), mycophenolate mofetil (MMF) and rituximab (RTX) is all associated with improvement in this established but relatively early SSc-ILD cohort across several pivotal trials.^[38]^

Conversely, interleukin 6 (IL6) appears to be important in early stage less extensive disease. This is supported by observational studies showing that serum IL6 levels predict future decline and worse outcome in those with limited extent ILD but not extensive disease where other non-IL6 dependent mechanisms may be more important to progression.^[39]^ This hypothesis was tested in the faSScinate and focuSSced clinical trials. Both studies showed a remarkable impact on progression of SSc-ILD.^[40,41]^ This was especially robust in the phase 3 trials and supported by quantitative CT analysis with improvement radiologically in all parameters of ILD on tocilizumab (TCZ) and decline on placebo that exceeded group level minimally clinically important difference for that evaluation method.^[40]^ Overall, the mild ILD in these early cases with elevated acute phase proteins was almost completely attenuated by TCZ. There were persuasive parallel experimental medicine studies of explant skin fibroblasts that found almost complete reversal of the profibrotic phenotype of activated fibroblasts.^[42]^ These better reflected clinical outcome in the lung than skin, where only a trend of benefit was shown in with clinical trial of TCZ, it aligns with the view that activated fibroblast drive early progression of SSc-ILD. This population of cells may be less relevant in later more established SSc-ILD. Recent post hoc analyses have shown that ATA+ cases drive the group level effect on forced vital capacity (FVC) in the phase 3 TCZ trial.^[43]^ Interestingly IL6 levels, in this cohort of cases all with increased IL6, did not predict greater response. This suggests that IL6 comes from multiple compartments and in those with very high levels perhaps it reflected other organs such as skin rather than lung fibrosis.

Post hoc analysis of the trial looking at more established SSc-ILD have also been informative. From the SENSCIS clinical trial of nintedanib which permitted background MMF treatment, there has been demonstration of additive benefits for immunosuppression and antifibrotic treatment.^[44,45]^ In addition, the link between extent of ILD on CT scan and risk of progression is clearly shown in the study cohort.^[46]^ MMF attenuates progression in milder cases but antifibrotic therapy seems to have more effect in extensive disease. Combination treatment decouples the link between extent of ILD and progression over 52 weeks highlighting the disease modifying effect and supporting the notion that combinatorial treatment approaches may be especially effective. Interestingly there is numerically a greater effect of Nintedanib on SSc-ILD cases that are ATA negative again supporting the concept that separate drivers form immuno-inflammation are key to later stage progression of extensive SSc-ILD.^[47]^ This is important because this is the phase of disease linked to greatest mortality either directly from SSc-ILD or due to associated group 3 pulmonary hypertension.

Clinical trials have also provided a platform for discovery of biomarkers and molecular surrogates in homogenous well characterised patients. The predicted role of pneumoprotein in severe or progressive SSc-ILD in trials and cohorts provides additional support to a role for alveolar epithelial injury in driving SSc-ILD.^[48]^

## Overarching Concepts

In SSc-ILD, the lung fibrosis is developing in the context of a multi system fibrotic disease, and it is likely that some similar mechanisms operate across different compartments. Nevertheless, a challenge to understanding the disease is elucidating what determines which organs are most specifically affected and the timing of the affect. As outlined above immune-inflammatory mechanisms appear to be very important in the early progressive phase of interstitial lung disease but may play a less prominent role at later stages. Understanding the drivers of fibrosis in other systems in SSc is likely to be informative.

There have been comparative biology studies looking at lung fibroblasts and other components in SSc and comparing them with skin. The same drivers including TGFbeta, endothelin 1 and various other cytokines and chemokines have been linked to both skin and lung disease. There are also likely to be lung specific mechanisms perhaps particularly linked to the biology of pulmonary epithelium, the alveolus structure and to the intrinsic resistance of lung tissue to scarring which is central to its function for respiratory gas exchange. In addition, factors such as aspiration and infection are specifically relevant to triggering or amplifying lung damage and inflammation.

It is notable that other forms of lung injury may be more recoverable. This is better exemplified by the interstitial abnormalities that have been reported during the coronavirus disease (COVID-19) pandemic which often show resolution over 12 to 18 months with persistence of the ILD related to systemic sclerosis.^[49]^ Similar findings have been observed for other viral infections again pointing to the potential for lung to respond differentially to triggers of injury and interstitial abnormality.

## Integrative Mechanisms and Immunopathogenesis

Cellular crosstalk and the interplay between the cellular components of lung and extracellular matrix are likely to be important in the pathogenesis of SSc-ILD. Endothelial and epithelial cell injury have been demonstrated to precede inflammatory infiltrate and fibrosis. This results in the activation of an immune response including cytokine release, up-regulation of pro-fibrotic mediators and recruitment of fibroblasts and fibrocytes.^[50]^ Dysregulated TGFbeta results in fibroblast proliferation and differentiation along with extracellular matrix production and deposition.[51] The WNT/β-catenin pathway has been shown to stimulate fibroblasts to myofibroblasts with suggestions WNT and TGFbeta may create a reciprocal loop sustaining myofibroblast activation.^[52,53,54]^ Derived myofibroblasts are thought to be the key effector cell of fibrosis in SSc-ILD.^[55]^

The adaptive and innate immune system are implicated in propagating fibrosis from a variety of studies. There is substantial interest in macrophage dysfunction and SSc, in particular and abnormal polarisation towards a pro fibrotic phenotype for macrophages.^[51]^ It seems inflammatory cells may be more important in initiating lung injury but may also have a physiological or pathophysiological role in the resolution of fibrosis that would usually occur after injury. Simple associations between monocyte count and severity or progression of lung fibrosis provide additional support as in the recently published study from the phase 3 trial of tocilizumab in systemic sclerosis.^[56]^

The adaptive immune system is likely to be important particularly through immunopathogenic mechanisms and multiple cell types are implicated. Increased activated natural killer (NK) cells, and a shift from resting to effector tissue resident CD8 T cell have recently been found in SSc-ILD lungs with these cells implicated in the pathogenesis of other autoimmune diseases although the mechanisms remain unclear.^[57]^

There is considerable interest in the role of B cell dysfunction particularly as targeting B cells therapeutically with rituximab has been promising.^[58]^ Ongoing clinical trials in SSc-ILD are looking at other B cell target approaches including CD19 chimeric antigen receptot T cell (CAR-T) therapy and belimumab. B cells have been demonstrated to have increased activation markers with increased CD19 levels particularly in ATA positive patients.^[59]^ Skin from early SSc has been found to have an innate and adaptive inflammatory profile including B cell signatures which may initiate the fibrotic process.^[60]^ B cells interact with immune cells and non-immune cells both through cytokine production and other mechanisms including antibody production. ATA producing CD27^+^ B cells with a low affinity for topoisomerase I have been found to have a higher frequency of anti-inflammatory cytokine production whereas cells with a high affinity for topoisomerase I produced increased pro-inflammatory cytokines including IL6 and IL23. ^[61]^ Consistently abnormal B cell homeostasis has also been found in SSc and of particular interest the transitional B cells seem to be dysregulated in systemic sclerosis. These transitional B cells may over produce interleukin 6, especially in those patients with anti-topoisomerase-1.^[62,63]^ These B cell abnormalities would start to provide a linking mechanism between the auto antibody subtype and the early development of severe lung fibrosis that has been seen both clinically and in clinical trial cohorts. Despite the low frequency of circulating autoreactive CD4^+^ T cells in SSc, elevated Topoisomerase-1 specific CD4 T cells restricted among ATA positive patients with Th17 proinflammatory phenotype was reported to associate with SSc-ILD progression.^[64]^ This opens up the possibility of B and T cell collaboration and disruption of this network may present a future therapeutic strategy.

Increased lung stiffness due to extracellular matrix deposition is known to be important in the propagation of lung fibrosis and likely play a role in subsequent progression of SSc-ILD. Fibroblasts exposed to high levels of stiffness have a more profibrotic phenotype and TGFbeta activation is also increased with increased lung stiffness.^[37,65]^ The change in lung mechanics along with recurrent lung injury and resulting activation of the immune system are probable factors in the cyclical activation of pathways resulting in further lung fibrosis.

**Figure 2:**
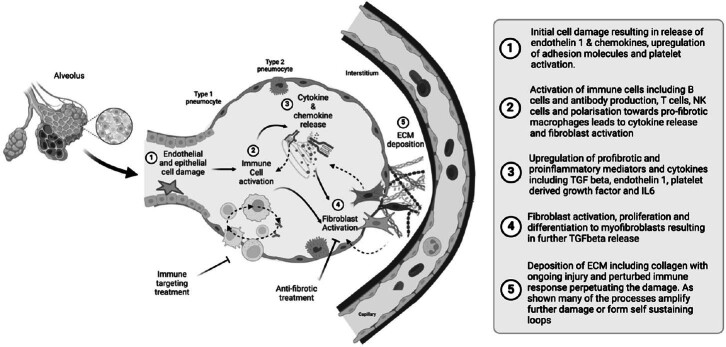
Cellular mechanisms of SSc-ILD. IL6, Interleukin 6; ECM, extra cellular matrix (Figure created with BioRender.com).

A summary schematic of the cellular mechanisms relevant to SSc-ILD immunopathogenesis is shown in Figure 2.

## Relevance to Clinical Practice and Trials

Understanding pathogenesis is important because it links directly to the concept of precision or stratified medicine. Whilst aetiology maybe more difficult to tackle in the context of established SSc there maybe opportunities in the future to prevent or intervene at a very early stage in ILD. Notable is the evidence from the very early diagnosis of systemic sclerosis (VEDOSS) study that even at a very early stage before patients fulfilled the classification criteria for systemics process there is evidence of interstitial lung abnormality and pulmonary function test (PFT) deficits that may suggest very early lung disease.^[66]^ Identifying these high-risk cases may offer new opportunities for prevention of lung fibrosis. Prognostic markers can predict the risk of progression and there are already some established markers such as interleukin 6 acute phase markers and the pneumoprotein Krebs von den Lungen-6 (KL6). Prognostic markers will help guide decision making regarding who to treat reducing unnecessary treatment burden but hopefully preventing progression. Predictive markers are also important but are dependent on defining therapeutically effective drugs and this is an area for future research.

## Future Challenges and Opportunities

The integration of epidemiological and genetic studies together with preclinical models to test hypothesis of pathogenesis *in vivo* have allowed us to develop much more comprehensive and relevant hypothesis and mechanisms to explain SSc-ILD. A key observation is that there are likely to be different phases and drivers of the disease through its natural history that differ between different subgroups defined by the extent of skin involvement or by autoantibody profile. This already allows treatment decisions to be made that incorporate these aspects of the disease and help in interpreting clinical trial results.

The therapeutic space of SSc-ILD is an area of intensive clinical trial activity and some drug trials are testing established drugs that would soon be available for clinical use if the trials are positive. Conversely other exciting trials are testing novel potential pathogenic mechanisms, and this will not only give more insight into the pathogenic drivers and mechanisms of SSc-ILD but also give the potential to move to the next level of combination therapies targeting different and complementary pathogenic disease mechanisms. There is great reason to be optimistic but the challenges of performing effective clinical trials and translating the results of those trials into accessible therapies for patients should not be underestimated.
